# Case Report: Solitary scalp metastasis after surgery for invasive ductal carcinoma of the breast

**DOI:** 10.3389/fonc.2025.1709244

**Published:** 2025-12-09

**Authors:** Chong Cheng, Tiejun Wang

**Affiliations:** 1 Department of Biotherapy, West China Hospital, Sichuan University, Chengdu, Sichuan, China; 2 Breast Cancer Center, Hubei Cancer Hospital, Tongji Medical College, Huazhong University of Science and Technology, National Key Clinical Specialty Discipline Construction Program, Hubei Provincial Clinical Research Center for Breast Cancer, Wuhan Clinical Research Center for Breast Cancer, Wuhan, Hubei, China

**Keywords:** breast cancer, scalp metastasis, hormone receptor, CDK4/6, endocrine therapy

## Abstract

**Background:**

Breast cancer is one of the most common malignancies among women, and more than 90% of breast cancer-related deaths attributed to metastasis. Cutaneous metastases are relatively uncommon, and scalp involvement is exceedingly rare. Because of its atypical clinical presentation, scalp metastasis is often recognized and diagnosed only after a delay.

**Case presentation:**

We report the case of a 45-year-old woman diagnosed with invasive ductal carcinoma (IDC) of the right breast with ipsilateral axillary lymph node involvement (clinical stage cT4bN3M0). The patient received six cycles of neoadjuvant chemotherapy and achieved a partial response (PR) according to RECIST 1.1 criteria. Subsequently, she underwent a right simple mastectomy with axillary lymph node dissection. Pathology demonstrated a Miller–Payne grade 3 response, with metastases identified in all 13 dissected axillary lymph nodes (13/13). Immunohistochemistry (IHC) revealed estrogen receptors (ER) and progesterone receptors (PR) positivity, human epidermal growth factor receptor-2 (HER2) expression of 0, and Ki-67 expression of 20%. She subsequently received adjuvant radiotherapy and endocrine therapy. Surveillance imaging during follow-up showed no evidence of recurrence or distant metastasis. In December 2023, the patient developed a painless, round, skin-colored nodule on the left frontal scalp, accompanied by diffuse right periorbital edema and headache. Over the following year, she was evaluated in dermatology, neurosurgery, and oncology clinics. However, breast cancer metastasis was not initially suspected, resulting in misdiagnosis and delayed treatment. In April 2025, fine-needle aspiration of the scalp nodule confirmed metastatic carcinoma. IHC showed ER and PR positivity, HER2 expression of 1+, and Ki-67 expression of 35%. No additional metastatic lesions were identified. The patient was started on systemic therapy with fulvestrant plus dalpiciclib, and after four cycles, she achieved marked regression of the scalp lesion along with resolution of periorbital edema. The most recent examination, however, detected meningeal and calvarial metastases. Consequently, the patient received localized radiotherapy to these sites while continuing the original treatment protocol.

**Conclusion:**

This case highlights the diagnostic challenges of atypical scalp metastases in breast cancer and underscores the importance of early detection and prompt initiation of comprehensive treatment.

## Introduction

Breast cancer remains one of the most significant health challenges among women worldwide. Although its incidence remains high, advances in screening, early diagnosis, and systemic therapies have led to a gradual decline in mortality rates (1). Nevertheless, once distant metastasis occurs, the prognosis is poor, with a 5-year survival rate of only approximately 25% (2).

The most common sites of distant metastasis from breast cancer are the bone, lung, brain, and liver (3). In a large retrospective study of 4,020 patients, the findings demonstrated a 23.9% prevalence of skin metastases in female breast cancer patients (4). Among the molecular subtypes of breast cancer, triple-negative breast cancer (TNBC), although accounting for only about 15% of primary tumors, constitutes up to approximately 30% of cutaneous metastases, demonstrating a specific tropism for the skin (5). In contrast, the proportion of HR-positive/HER2-negative subtypes in skin metastases is generally consistent with their incidence rate, showing no particular propensity for such spread (6). Scalp metastasis represents an exceedingly rare form of cutaneous involvement, with a reported incidence of less than 1%, and it remains insufficiently characterized in the literature (7).

Here, we describe a case of hormone receptor–positive (HR+) invasive ductal carcinoma (IDC) of the breast that developed a solitary metastatic lesion in the left forehead skin after surgery, with no evidence of metastases at other sites.

## Case presentation

A 45-year-old woman with a history of primary ovarian insufficiency, diagnosed at the age of 35, had been receiving oral estradiol valerate combination tablets for hormone replacement therapy. In 2017, a right ovarian cyst was detected and surgically excised, after which hormone replacement therapy was discontinued.

**Figure 1:**
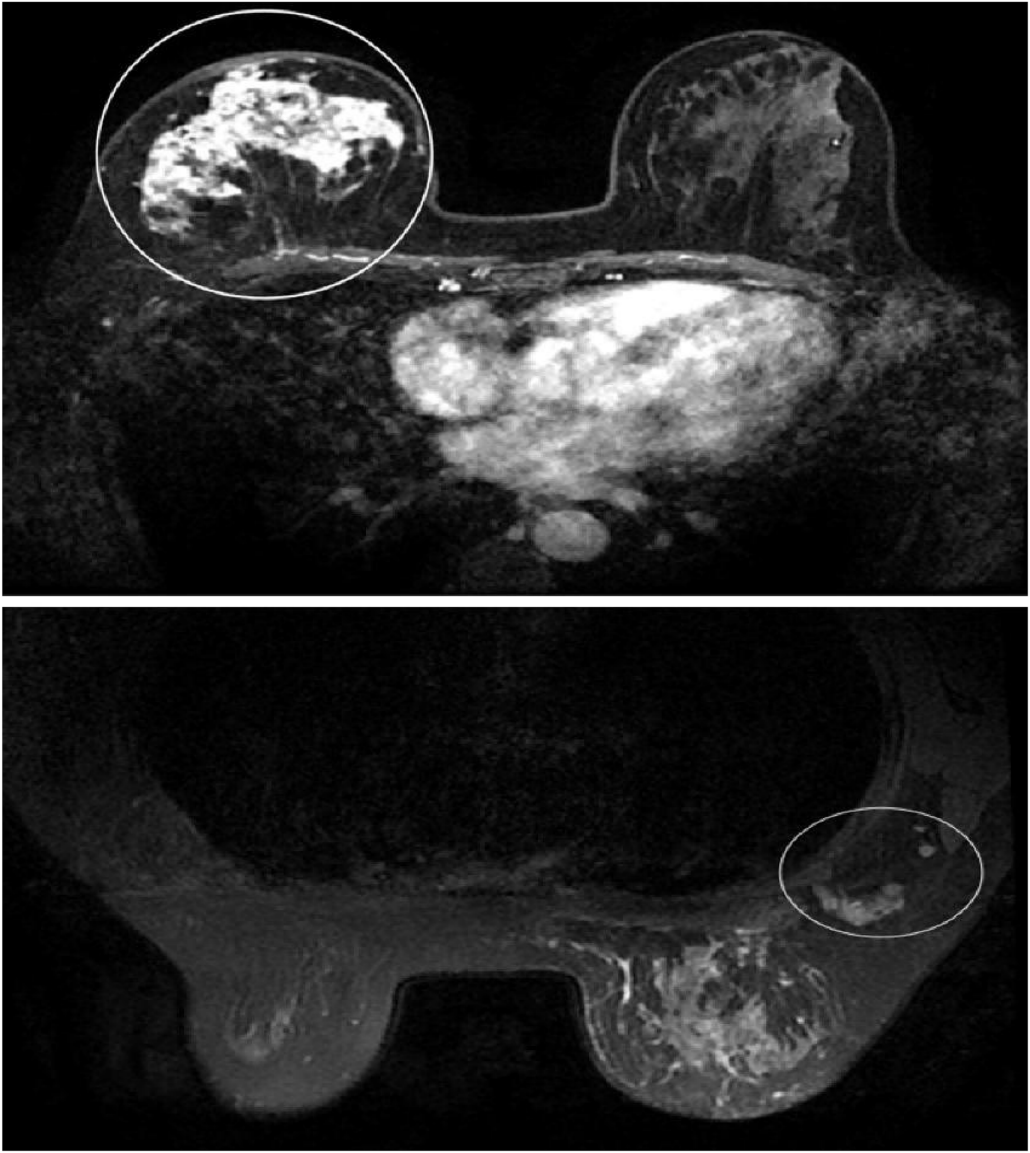
The baseline breast MRI revealed non-mass-like enhancement in the right breast, measuring 7.5× 5.2 × 8.4 cm. Multiple enlarged lymph nodes are visible in the right axilla, with the largest measuring approximately 1.5 cm in short diameter.

**Figure 2:**
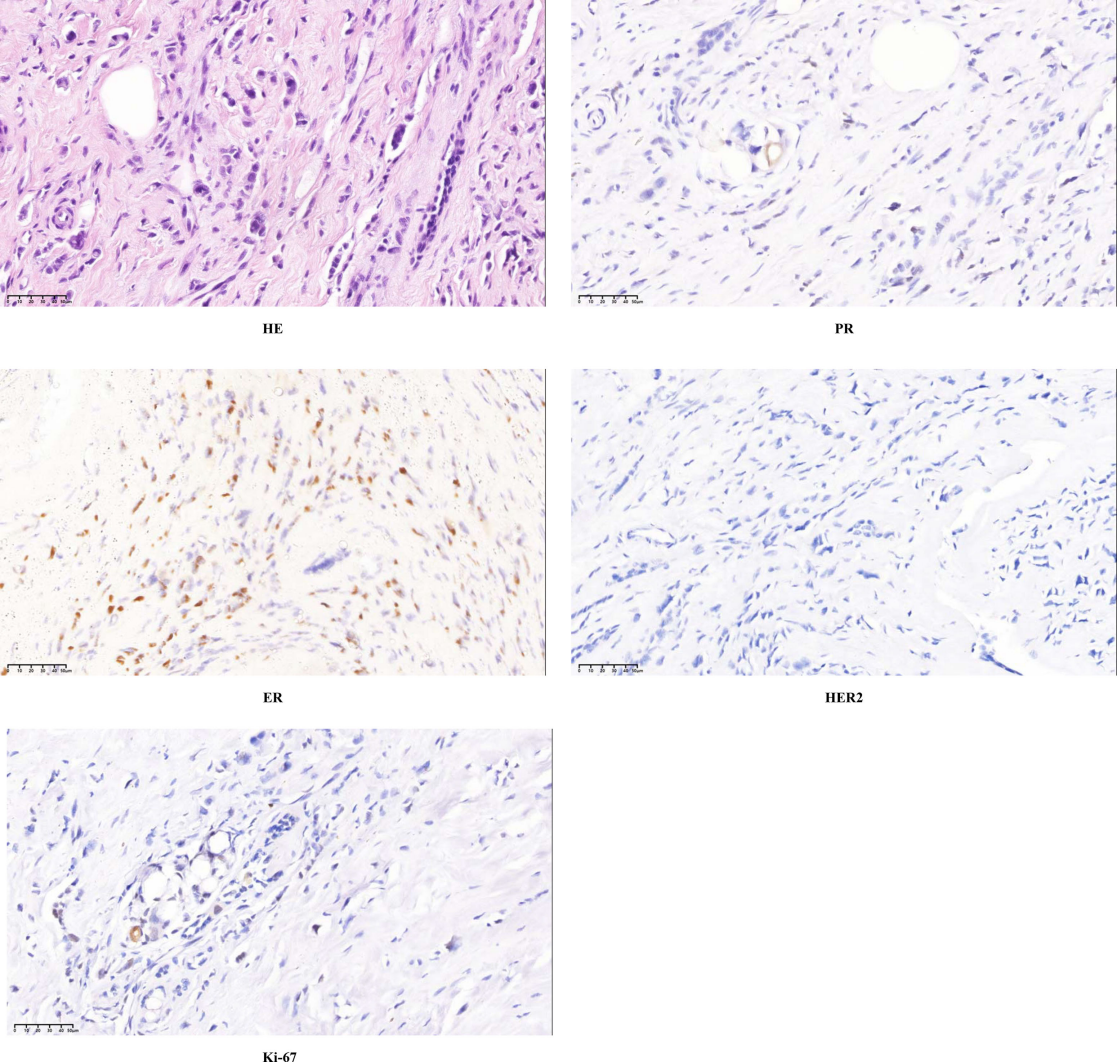
Preoperative biopsy indicated invasive ductal carcinoma: HE staining, ER (40%), PR (-), HER2 (0), Ki67 (10%). HE staining, Hematoxylin-Eosin staining; ER, estrogen receptor; PR, progesterone receptor; HER2, human epidermal growth factor receptor-2; Ki67, nuclear-associated antigen Ki67.

In May 2019, the patient palpated a mass in her right breast. On physical examination, an irregular, firm mass measuring approximately 8×6 cm was identified at the 2 o’clock position of the right breast. The lesion exhibited ill-defined borders, limited mobility, and was accompanied by multiple enlarged right axillary lymph nodes. Breast magnetic resonance imaging (MRI) demonstrated non-mass-like enhancement of the right breast, measuring 7.5×5.2×8.4 cm, along with multiple enlarged right axillary lymph nodes, the largest having a short-axis diameter of 1.5 cm (**Figure 1**). In October 2019, fine-needle aspiration biopsy confirmed invasive breast carcinoma. Immunohistochemistry (IHC) revealed estrogen receptor (ER) positivity at 40%, progesterone receptor (PR) negativity, human epidermal growth factor receptor-2 (HER2) expression of 0, and Ki-67 expression of 10% (**Figure 2**). Fine-needle aspiration of right axillary lymph nodes at levels I and III confirmed metastatic carcinoma cells. Contrast-enhanced chest Computed Tomography (CT), abdominal ultrasound, contrast-enhanced head MRI, and whole-body bone single photon emission computed tomography (SPECT) showed no evidence of metastatic disease elsewhere. The clinical stage was determined as cT4bN3M0.After that, the patient underwent six cycles of neoadjuvant chemotherapy with docetaxel (75mg/m^2)^ plus epirubicin (75mg/m^2)^. Post-treatment imaging demonstrated a partial response (PR) according to RECIST 1.1 criteria (8).

On March 17, 2020, the patient underwent a right simple mastectomy with axillary lymph node dissection and skin flap reconstruction under general anesthesia. Pathology revealed grade 3 invasive ductal carcinoma with a Miller-Payne grade 3 pathological response and metastases in all 13 dissected axillary lymph nodes (13/13). IHC demonstrated ER positivity at 90%, PR positivity at 70%, HER2 expression of 0, Ki-67 expression of 20%. The postoperative pathological stage was determined as ycT4bN3M0. From July 6 to August 11, 2020, the patient received chest wall radiotherapy (50 Gy in 25 fractions), and subsequently she initiated adjuvant endocrine therapy with letrozole 2.5 mg daily combined with goserelin acetate 3.6 mg every 28 days. Follow-up imaging studies performed every 2–3 months showed no evidence of recurrence or distant metastasis. The modalities included mammography, breast ultrasound, contrast-enhanced chest CT, abdominal ultrasound, contrast-enhanced head MRI, and SPECT bone scan.

**Figure 3:**
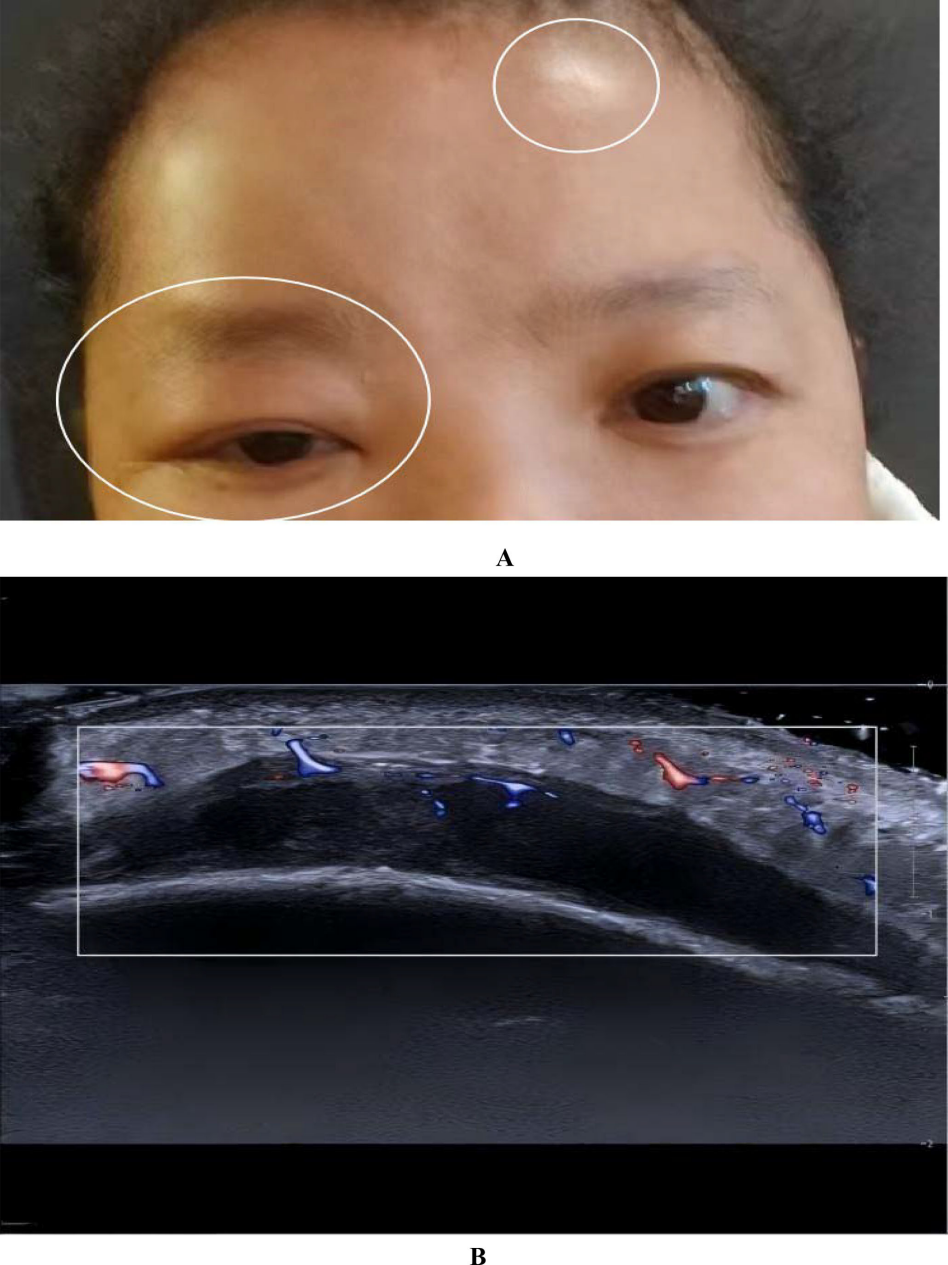
A single small nodule is present on the left frontal area, approximately 1×2 cm in size, white in color, with relatively clear borders. The surface shows no obvious ulceration or purulent discharge. Simultaneously, redness and swelling appeared around the right eyelid **(A)**. The superficial tissue ultrasound suggested thickening of the subcutaneous layer in the frontal area, with a maximum thickness of approximately 4.9 mm. The echogenicity was reduced, and punctate or linear blood flow signals were observed within **(B)**.

**Figure 4:**
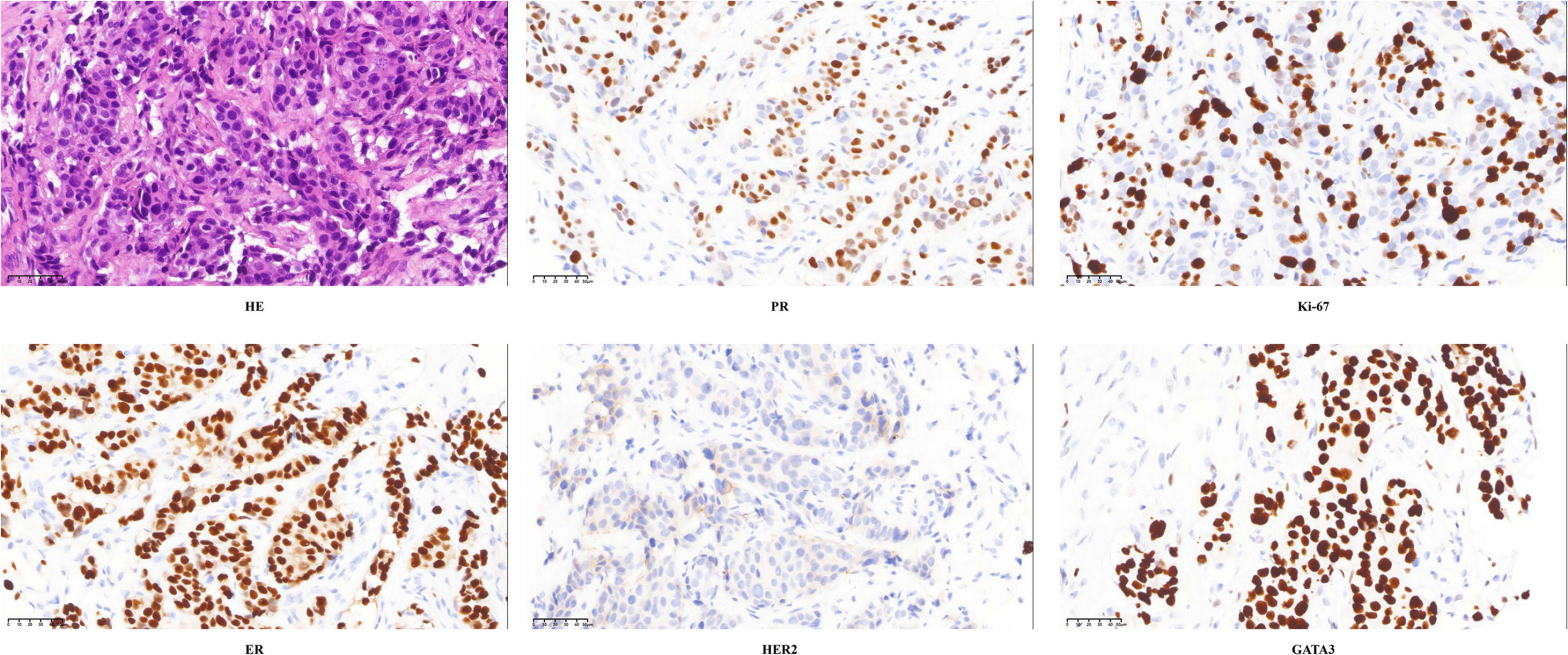
The pathological results of the left frontal nodule biopsy: HE staining, ER (>90%), PR (60%), HER2 (1+), Ki67 (35%), GATA 3(+). HE staining, Hematoxylin-Eosin staining; ER, estrogen receptor; PR, progesterone receptor; HER2, human epidermal growth factor receptor-2; Ki67, nuclear-associated antigen Ki67; GATA3, GATA 3 binding protein.

**Figure 5:**
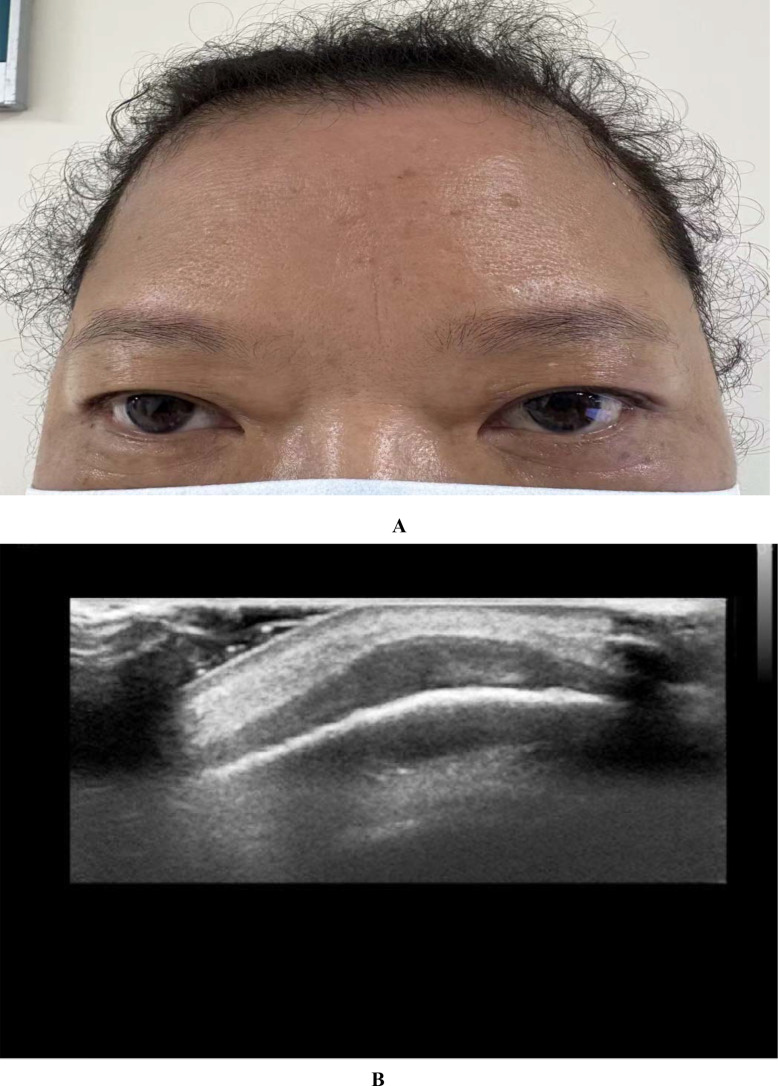
The palpable mass was almost completely resolved, and the edema around the right eyelid disappeared, with significant improvement in symptoms **(A)**. After 4 cycles of Fulvestrant combined with Darsalizumab treatment, a follow-up superficial tissue ultrasound showed thickening of the subcutaneous layer in the left frontal area, with a maximum thickness of approximately 2.9 mm. The echogenicity was slightly reduced, and no significant blood flow signals were observed **(B)**.

**Figure 6:**
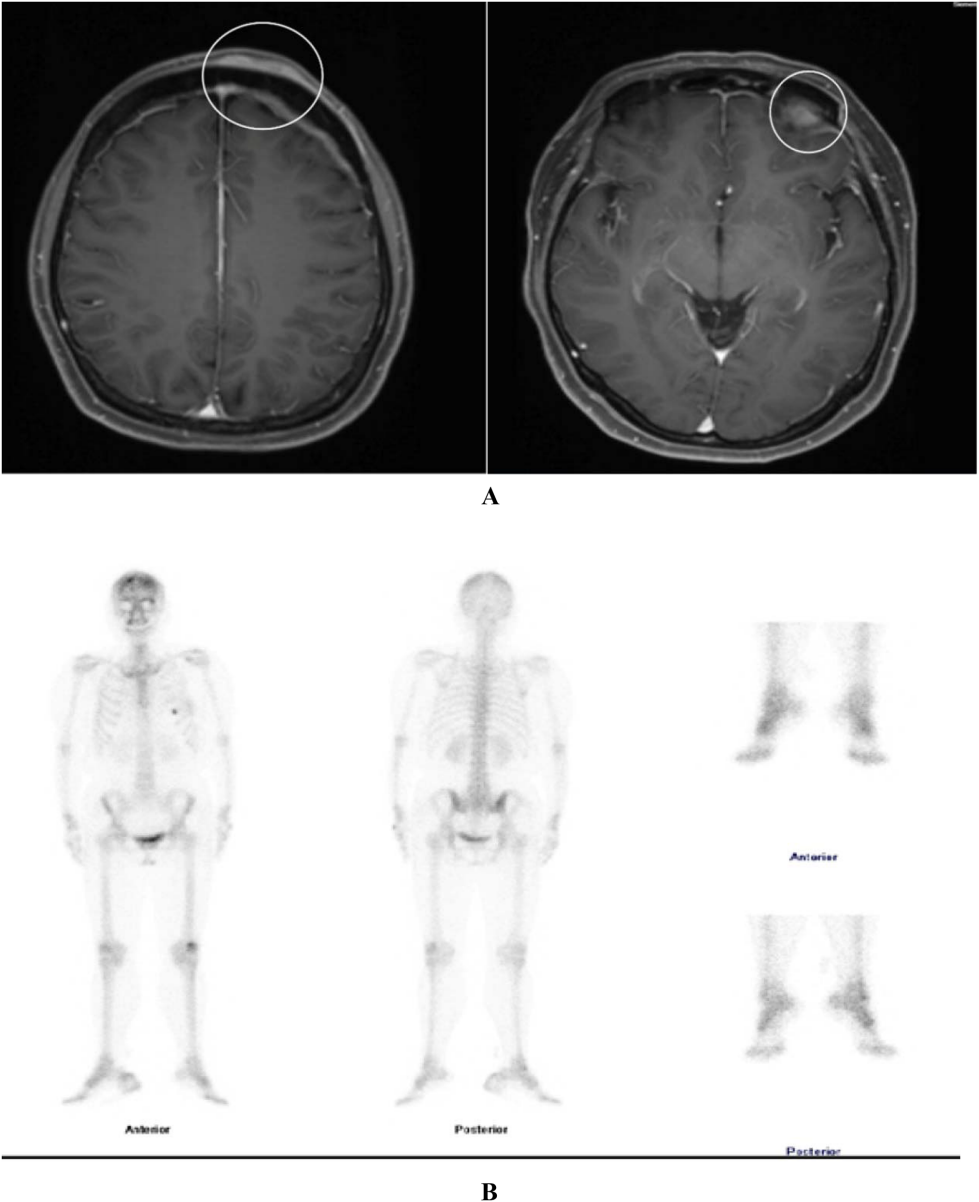
Contrast-enhanced MRI of the head (**A**) reveals a linear and patchy shadow in the left frontal subcutaneous area, a localized small nodular shadow under the inner plate of the left frontal skull, and thickening of the left frontal meninges with more pronounced enhancement compared to the contralateral side. Whole-body bone SPECT (**B**) reveals irregular hypermetabolic foci in the frontal bone, suggestive of osseous metastasis, with significantly decreased metabolic activity compared to prior findings.

In December 2023, the patient developed a solitary, painless nodule measuring approximately 1×2 cm on the left frontal scalp, with intact overlying skin, accompanied by diffuse right periorbital edema (**Figure 3A**). In January 2024, the patient was referred to dermatology, neurosurgery, and oncology clinics due to progressively worsening symptoms. Initially, the lesion was considered unrelated to her breast cancer, with differential diagnoses focusing on benign conditions such as epidermoid cyst, lipoma, dermatofibroma, and angioedema. Empirical anti-inflammatory and symptomatic treatment provided only transient relief, with symptoms recurring shortly thereafter. In February 2025, the patient’s serum carcinoembryonic antigen (CEA) level was mildly elevated at 5.93 ng/mL (previously within normal limits). Ultrasonography demonstrated a reduction in scalp lesion thickness to 4.9 mm (**Figure 3B**). Positron emission tomography (PET) was recommended to assess systemic disease, but the patient declined due to financial constraints. Ultrasonography of the surgical site showed no evidence of local recurrence, and contrast-enhanced head MRI, contrast-enhanced CT, bone scan, and abdominal ultrasound revealed no distant metastases. In April 2025, fine-needle aspiration of the scalp lesion confirmed metastatic carcinoma. IHC demonstrated ER positivity >90%, PR positivity at 60%, HER2 expression of 1+, and Ki-67 expression of 35% (**Figure 4**). On April 30, 2025, the patient initiated systemic therapy with fulvestrant (500 mg administered on days 1, 15, and 29, then monthly thereafter) in combination with dalpiciclib (150 mg once daily for 21 consecutive days, followed by a 7-day treatment-free interval, in 28-day cycles). After four cycles, regression was observed, with the lesion thickness reduced to 2.9 mm and complete resolution of periorbital edema (**Figure 5A**, **Figure 5B**). However, the patient subsequently developed significant headache. contrast-enhanced head MRI and SPECT bone imaging revealed metastases to the meninges and frontal bone (**Figure 6A**, **Figure 6B**). While continuing the original endocrine therapy combined with a CDK4/6 inhibitor, the patient has initiated radiotherapy for the intracranial metastases. Radiation simulation has been completed, and treatment planning is currently underway.

**Figure 7:**
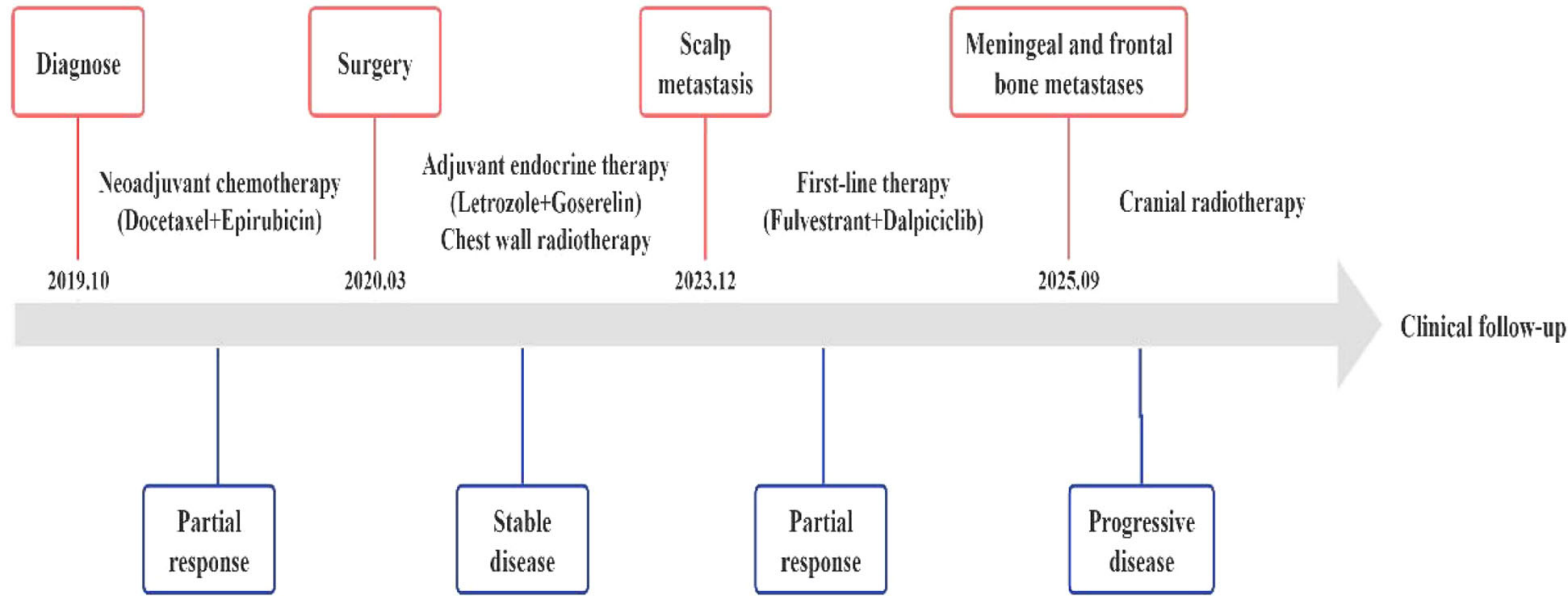
The detailed treatment timeline is shown in the figure above.

The detailed treatment timeline is shown in the figure below (**Figure 7**).

## Discussion

Breast cancer-related cutaneous metastases occur through distinct patterns, primarily categorized as local (approximately 86%) and distant (approximately 14%) spread (9). Local metastases frequently arise through regional lymphatic dissemination near the primary tumor, commonly manifesting on the chest wall (10). In contrast, distant cutaneous involvement such as scalp metastases may occur via hematogenous spread or retrograde flow through the cervical-occipital lymphatic network (11). The molecular profile of the tumor, including hormone receptor and HER2 status, significantly influences metastatic tropism, while interactions between circulating tumor cells and the cutaneous microenvironment further support the colonization process (12). We have summarized the published cases of breast cancer with scalp metastasis (**Table 1**).

**Table 1:** Summary of reported cases of scalp metastasis in breast cancer.

Date	Primary histology	Primary subtype	Sequence of scalp metastasis	Scalp location	Clinical presentation	Metastasis subtype	Local recurrence	Other metastases	Treatment	Outcome	Reference
2025	IDC	Luminal HER2-negative	Recurrence	Frontal scalp	Firm, non-pulsatile, mildly tender; overlying skin intact without ulceration, discharge, or inflammation.	Luminal HER2-negative	/	Calvarial metastasis	RT: 30 Gy/10 fx; 1L: Paclitaxel 80 mg/m²;2L: Capecitabine 1000 mg/m².	Died 10 months after metastasis diagnosis	(11)
2025	Mammary ductal carcinoma	/	Recurrence	Posterior mid-parietal scalp	Solitary, well-circumscribed, pink, pearly, telangiectatic papule.	/	yes	Mediastinum, left hilum, liver, and numerous bony metastases.	Fusiform excision of the lesion.	/	(17)
2024	IDC	Luminal HER2-negative	Recurrence	Top of the scalp	A well-defined, red, elevated mass.	/	yes	Adrenal gland, kidney, brain and multiple bone metastases.	Radiation treatment for bone metastases;capecitabine; albumin-paclitaxel.	Stable condition	(7)
2024	IDC	Luminal HER2-negative	Recurrence	Top of the scalp and left occipital region	Flat hard, well-demarcated erythematous lesion without ulcerations.	Luminal HER2-negative	yes	Right axillary and left supraclavicular nodal metastases; multiple bone metastases.	Single-agent fulvestrant; eight cycles of salvage chemotherapy (paclitaxel and capecitabine).	Died after salvage chemotherapy.	(16)
	Invasive mucinous carcinoma	Luminal HER2-negative	Concurrent	Occipital scalp region	A 2.0 × 0.5-cm non-mobile non-erythematous elevated poorly demarcated nodule.	Luminal HER2-negative	/	Right axillary lymph nodes, 12th thoracic spine and the 1st lumbar spine metastases.	Six cycles of docetaxel, epirubicin, and cyclophosphamide; goserelin; right salpingo-oophorectomy; anastrozole.	Stable condition	
2024	Invasive lobular carcinoma	Luminal subtype	Recurrence	Left parietal area	A pinkish-hued, 2 cm-sized nodular lesion, accompanied by localized alopecia.	/	/	No	Excision of the scalp lesion; anastrozole.	Stable condition	(13)
2023	IDC	Luminal HER2-negative	Recurrence	Left frontal area and left parietal region	Erythematous, firm, non-mobile, and non-tender of three scalp nodules.	Luminal HER2-negative	yes	left axillary lymph node, spine, ribs, liver and both lungs.	Did not receive any treatment for scalp metastases.	Progressed to septic shock before she died.	(14)
2020	Invasive breast cancer	Luminal HER2-negative	Recurrence	Top left side of the scalp	Immobile, hardened, skin-colored, and without hair, and pressing on it resulted in pain and a diameter of 1.5 × 2.5 cm.	Luminal subtype	/	Right upper lobe bronchial wall, left supraclavicular fossa, and dorsal subcutaneous area.	Surgical removal of the scalp mass	/	(15)
2019	IDC	Luminal HER2-negative	Recurrence	Left temporal region	A 3-cm, movable, non-ulcerated nodule with normal overlying skin.	Luminal HER2-negative	/	no	RT: 3000 cGy/10 fx; Exemestane 25 mg daily.	Remained tumor-free through the last follow-up, with disease-free survival of one year.	(25)
2018	Occult breast carcinoma	/	Initial symptom	Left frontal scalp	Skin-colored, nontender, hard, immobile, and measured 2.5 cm.	/	/	No	Four cycles of adriamycin and cyclophosphamide; four cycles of docetaxel; surgical removal and graft of the scalp mass; radiation therapy; tamoxifen.	Stable condition	(23)
2017	Occult breast carcinoma	/	Initial symptom	Left parietal scalp	/	Luminal subtype	/	No	Re-excision of margins; anastrozole.	Unknown	(24)
2011	IDC	Luminal HER2-negative	Recurrence	Unknown	A reddish, indolent nodule of the scalp 5 mm in diameter with local alopecia.	Luminal HER2-negative	/	Second primary IDC of right breast; multiple pulmonary and ipsilateral axillary lymph node metastases.	Ablatio mammae right; one cycle of paclitaxel and bevacizumab.	Died due to sepsis.	(21)
2007	IDC	/	Initial symptom	The left temporo-parieto-occipital region	Infiltrative subcutaneous tumor beneath intact skin with tender, minimally bleeding ulcer.	Luminal HER2-negative	/	Right hilum, lungs, subpleural regions, liver, and cranial vault.	Palliative therapy	Unknown	(22)

IDC, invasive ductal carcinoma; HER2, human epidermal growth factor receptor-2; RT, radiotherapy.

In the present case, the patient developed a small, skin-colored nodule on the left frontal scalp, which initially posed diagnostic challenges due to its nonspecific appearance. Although the patient also presented with right eyelid edema, potentially related to impaired lymphatic drainage or occult infiltration, the final diagnosis of scalp metastasis was confirmed histopathologically. This clinical course aligns with previous reports describing scalp metastases as typically presenting as solitary or multiple nodules, though variable morphologies such as erythematous plaques or alopecia neoplastica have also been documented (7, 13–19). Notably, Tomasini et al. described a case of histiocytoid breast carcinoma with eyelid metastasis presenting as persistent swelling, reinforcing that any new cutaneous or adnexal lesion in patients with a history of breast cancer warrants thorough investigation to exclude metastasis (20).

Cutaneous metastases in breast cancer often coincide with systemic dissemination (7, 11, 14–17, 21, 22), though rare cases present as the initial sign of occult malignancy (23, 24). Solitary scalp metastasis from IDC is exceptionally uncommon. Only one comparable case has been reported in which the patient remained disease-free after surgical excision and local radiotherapy (25). By contrast, our patient did not undergo local treatment and subsequently developed calvarial and meningeal metastases following systemic therapy, highlighting the potential aggressiveness of this presentation even under active systemic management.

For patients with HR+/HER2− advanced breast cancer, endocrine-based therapy remains the cornerstone of systemic treatment. CDK4/6 inhibitors such as palbociclib, abemaciclib, and ribociclib are well-established in combination with aromatase inhibitors in the first-line setting (26–28). In this case, the patient received fulvestrant plus dalpiciclib, a selective CDK4/6 inhibitor shown to significantly prolong progression-free survival (29), and achieved initial partial response. Nonetheless, disease progression involving intracranial sites occurred subsequently.

The anatomical proximity between scalp and intracranial structures poses a particular risk for neural invasion in cases of scalp metastasis (11). This clinical trajectory underscores the importance of considering combined-modality strategies incorporating local therapy, for example surgery or radiation, alongside systemic treatment in patients presenting with scalp involvement. This approach may help mitigate the risk of intracranial extension and improve long-term disease control.

## Conclusion

We report a rare case of solitary scalp metastasis from IDC following surgery. The atypical clinical presentation resulted in misdiagnosis and delayed initiation of appropriate therapy. Clinicians should maintain a high index of suspicion for metastatic disease when new, unexplained cutaneous lesions in breast cancer survivors. Early identification and timely pathological confirmation are crucial for initiating effective systemic therapy promptly. Given the high risk of intracranial progression, a rational combination treatment approach should be considered.

## Data Availability

The original contributions presented in the study are included in the article/supplementary material. Further inquiries can be directed to the corresponding author.
